# An Inquiry, Critical and Experimental, into the Pathology of Fever

**Published:** 1853-09

**Authors:** N. S. Davis

**Affiliations:** Professor of Pathology, Practice of Medicine, and Clinical Medicine, in Rush Medical College; and one of the Physicians to the Ill. Gen. Hospital


*An Inquiry, Critical and Experimental, into the Pathology of Fever.* By N. S. Davis M. D. Professor of Pathology, Practice of Medicine, and Clinical Medicine, in Rush Medical College; and one of the Physicians to the Ill. Gen. Hospital.

[Continued from Page 256.]

Morbid appearances are often found in other parts of the body than those already enumerated, such as the Œsophagus, the Pancreas, the Mesentery, and the muscles generally, but they are neither sufficiently constant nor peculiar in their appearance to require particular notice. At the last annual meeting of the American Medical Association, Dr. Campbell, of Georgia, presented a paper or report on Typhoid Fever, in which he claims that the disease has its special origin and seat in the ganglia of the sympathetic nerve. But how far he has verified the existence of disease in those ganglia and plexuses, by actual post-mortem examinations, I do not know; the annual volume of transactions, containing the report, not having yet passed through the press.* The foregoing brief synopsis of the morbid anatomy of continued fever, is sufficient to indicate the extent and nature of those changes which take place during the progress of that class of diseases; and it is worthy of remark, that here, as in fevers of the periodical class, the more malignant and rapidly fatal the progress of the disease, in any given case, the less are the traces of the disease found in the solid structures of the body after death. In a large proportion of such cases, the only appreciable lesions are the dark color and fluid, or but partially coagulated state of the blood: its accumulation in the veins of the more vascular organs; and a general flabby or impaired state of the textures of the body. In the more protracted cases, we have, in addition to the altered state of the blood and the general passive venous congestion, far greater structural changes, especially in the mucous lining of the alimentary canal, the spleen, the lungs, and the heart. But what is the *nature* of these changes? Are they merely the results of inflammatory action, or do they result from an altered relation between the fluids and solids, involving the elementary vital properties, and essentially different from inflammation?

The answer to these questions must depend, in a great degree,

* See Appendix, Note D.

Reel 178-10-5 cont on the meaning which we attach to the word inflammation. If we restrict the application of that word to a specific and uniform morbid process, characterized by *active* local determination of blood, with increase of fibrin in the same, and tendency to induration or suppuration in the solids, then very few of the post-mortem phenomena of continued fever can be attributed to inflammation.

Or, if we extend the application so as to embrace the definition of Dr. Williams, viz.: "too much blood in a part with its motion, partly increased and partly diminished," we shall still find difficulty in many cases. For Dr. Williams evidently means by "motion partly *increased* and partly *diminished*," that the flow of blood to and around the inflamed part is increased, while its passage *through* is retarded or diminished, thereby inducing accumulation in the part. Hence he says in reference to his definition, that "it is easily demonstrated in the *strong pulse* of arteries, leading to an inflamed part, and in the stagnation of much blood in the part."

According to the views of this distinguished author, all three of the elements named are essential to constitute inflammation. The first, consisting of increased flow of blood *to* the part, distinguished it from simple *congestion;* while the second, consisting of retarded flow of blood *through* the part, equally distinguishes it from simple *determination* of blood; and the third, consisting of an accumulation of too much blood in the part, is a necessary consequence of the two preceeding. Are all these conditions of the circulation always, or even generally, present in the organs and tissues most frequently found diseased in connection with continued fevers? That the blood is in many cases retarded in its passage through certain organs, and accumulated in them in undue quantity, is abundantly shown by the engorged state of the vessels of the cerebral meninges, the depending portions of the lungs, the spleen, the mesentery, and portions of the mucous membranes. But that there is an increased flow of blood *to* these several organs, is by no means so certain. It is true, that Dr. Stokes, in his lecture on what he styles Ileitis or *Dothinenteritis*, which is, to some extent, at least, synonymous with the typhoid fever of others writers, speaks of the increased pulsation of the abdominal aorta and its branches, as an important diagnostic sign of inflammation of the bowels. In ordinary inflammation of the intestines, such pulsation is doubtless constantly present, and it may be easily detected in some cases of typhoid fever with strongly marked abdominal symptoms; but I have carefully noticed several cases, in which extensive ulcerations of the mucous lining of the ilium existed, without detecting any undue pulsation in the abdominal arteries during life. And though the tympanitic distension of the abdomen sometimes renders it difficult to appreciate the condition of the arteries within, yet I am sure that increased pulsation in those vessels, is not a constant symptom even in the most strongly marked cases of enteric typhoid fever. The condition of the arteries supplying the brain and lungs is much more readily examined; and while I have, in some cases, found the pulsation in both increased, in many others the beating of the carotids and the heart itself, has been feebler than natural. In the wards of the Hospital, especially, I have often met with cases, in which there was almost constant delirium of a passive, dull character, and dulness on percussion over the dependant part of the lungs, and at the same time the pulsations of the heart and carotids, though quick, were decidedly feeble. If these observations have been made with due care, they fairly authorize the conclusion that many of the local appearances of disease resulting from continued fever, lack that element of inflammation which consists in active increased determination of blood to the affected parts. But another element of inflammation, which the researches of Andral, Gavarret, Simon and others have led the profession to regard as essential as the conditions of the circulation described by Williams, consists in the rapid increase of fibrin and white corpuscles. And yet, all the analyses which I have been able to collect, as well as those made by myself, together with careful microscopic examinations, show both these constituents of the blood diminished rather than increased in all the forms of continued fever. Hence, if we give to the word *Inflammation* any definite meaning, or to the morbid processes to which it is applied, any constant elements, we must ascribe the local appearances of disease generally seen in the brain, lungs, heart, and abdominal viscera, after death from continued fever, to a process essentially different from true inflammation. By this remark I would by no means convey the impression that local *inflammation* never complicates continued fever; on the contrary, one of the pathological specimens, to which allusion has already been made, consisting of a portion of the ilium and mesentery taken from a patient dead of typhoid fever, presents not only very extensive ulceration in the glandular patches of the mucous membrane, and enlargement with softening of the mesenteric glands, but also two or three patches of unequivocal inflammation of the peritoneal membrane covering the enlarged mesenteric glands, with a well marked layer of coagulable lymph, partially organized into false membrane adhering to the inflamed surface. Equally unequivocal marks of genuine inflammation are sometimes seen in the lungs and brain. But these are certainly occasional occurrences, or exceptional cases rather than the ordinary phenomena presented after death from continued fever. Hence they should not be confounded with the venous engorgement of the membranes of the brain, the diffused redness of the inner surface of the heart and large bloodvessels, the black engorgement of the dependent portions of the lungs, the enlarged and disorganized condition of the spleen and mesenteric glands, the discolored and ulcerated condition of the mucous membrane of the intestines, and the softening of the tissues generally, which constitute the common *post-mortem* phenomena detailed by all writers on this subject: these are doubtless the result of what Dr. Drake has styled *Passive Hyperamia*, a morbid condition much more closely corresponding with the *congestion* of Dr. Williams, than with his description of inflammation. If it is true that the symptoms during life and the analysis of the blood, show that in very many cases presenting extensive local lesions after death, two of the well known and essential elements of inflammation are wanting, viz.: active determination of blood to the affected parts, and rapid accumulation of fibrin and white corpuscles, it is equally true that the local changes themselves do not generally correspond strictly with the changes well known to result from unequivocal inflammation in the same structures. These latter are thickening effusions of serum or plastic lymph on the membranous surfaces; hepatization in the lungs; induration and suppuration in the spleen, &c. While, as already remarked, the appearances left in these several structures after continued fever are, in a large majority of cases, those of simple venous engorgement, with enlargement and softening of texture. The alteration which has been most frequently mistaken for inflammation, is that state of the lung which Jenner calls "*lobular non*-granular consolidation." He describes it as follows, viz.: "externally, a portion of the lung in this condition has a mottled aspect; here and there are patches, varying in size from a single lobule to half or more of a lobe, of a deep bluish, chocolate, violet, or purplish slate color, bounded by a well-defined angular margin, crossed, if it includes more than one or two lobules; and mapped out into smaller patches by dull opague whitish lines. On closer inspection, the outline and the whitish lines intersecting the patches, are seen to be thickened interlobular septa. * * * * On section, the tissue corresponding to the dark patches is found to be of a deep purplish chocolate color, gorged with non-ærated bloody looking fluid, breaks down with little or no facility, nay, sometimes appears tougher than in health; has a uniform or nearly uniform section, i. e., *there is no appearance of granules, such are seen in the consolidated and non-consolidated state of so called vesicular pneumonia, etc.*"

Dr. Barlett remarks, in regard to this particular change in the lungs, that "it is quite unlike, in almost every respect, the second stage of inflammation, although the term *hepatization* has sometimes been applied to it." And he adds, "it is not indicated by any peculiar symptom during life."* This last expression may be critically correct, and yet I am stisfied that the symptoms during life, if not *peculiar* in their nature, are sufficiently so in their combination to prevent errors in diagnosis. I have not unfrequently met with cases of continued fever in an advanced stage of their progress, in which there was much dulness or torpor of mind, a leaden or purplish color of the prolabia, a short or enfeebled respiration, a quick and compressible or gaseous pulse, little or no cough, and generally no expectoration, though in some instances a paroxysm of coughing with the expectoration of a thin reddish mucous occurred at remote intervals. The patients complain of no pain in the chest and no dyspnœa, though if you request them

*See Bartlett on Fever, page 87. Ed. 3d.

to inspire a full breath, it is easy to see that they inflate the lungs but very imperfectly. Percussion over the infra-clavicular regions generally elicits no morbid sounds, but well marked dulness is found over some part of the lower lobe of one or both lungs.

Auscultation sometimes shows no other change than feebleness of the respiratory murmur, especially in those places presenting too great a degree of dulness; but in a far larger number of cases a mixture of the dry and moist ronchi are heard in the bronchial tubes, and a feebleness or entire suppression of the respiratory murmur over the lower and posterior regions of the chest, with increased vibration of voice. These symptoms and signs point with much certainty to the existence of increased density of some portion of the pulmonary tissue, and yet they do not include the true crepitant rale of the first, nor the coarse mucous ronchus and bronchophony with the red or purulent expectoration of the second stage of ordinary pneumonia. When recovery takes place in cases complicated with this consolidated state of portions of the pulmonary tissue, the convalescense is generally very tedious and protracted, characterized by a debilitated and short respiration continuing sometimes even after the patient has recovered a pretty good degree of flesh and apparent health.

In regard to the changes observed in the spleen, Dr. Bartlett coincides fully with M. Louis, in the opinion that they are not the result of inflammation. After giving some facts to corroborate this opinion, he adds: "In the present state of our knowledge, it is enough, perhaps, to say, that these alterations of the spleen, in typhoid, as well as in other fevers hereafter to be described, depend on some special and peculiar cause connected with the diseases in which they occur, the nature and operation of which are unknown to us; and further, that the lesions seem to be associated with that pathological element so obscure in its nature and causes, but so extensive and fatal in its results, to which the term *congestion* has been applied; and not with that other element to which the term *inflammation* has been applied."

The same remarks are certainly equally applicable to the ordinary changes observed in the brain and its membranes, the heart, and the liver. The only post-mortem appearances about which there is much room for controversy, are those observed in the mucous membranes of the stomach and intestines, and the glands in the mesentery.

The question as to how far any one or all of the changes observed in the mucous membranes after death are peculiar to and characteristic of *typhoid* fever, I shall not discuss in this place. These changes consist of simple softening of the membrane, of discoloration, and of ulceration. In some cases we see but one of these alterations, and in others we find them all in different parts of the same membrane.

The first, or softening, occurs frequently in the stomach, especially in the larger curvature and near the cardiac orifice. The same parts are generally darker red than natural, and sometimes they are deeply ecchymozed.

In another class of cases the softened membrane is both paler and thinner than in health. The same alterations of color and consistence are observed in the duodenum, jejunum and colon, though much less frequently than in the stomach. In all these parts small irregular ulcers are also occasionally observed. But the mucous membrane lining the lower half of the ilium is far more frequently the seat of disease than any other portion of the alimentary canal. The color of this portion varies from a bright blush of redness to a dark brown. Sometimes the membrane appears thickened, and the villi more prominent, giving the whole surface a *mammellated* appearance; in other cases, the thickening is confined to the aggregated glands of Pyer, causing them to appear in some places more prominent than the surrounding membrane, and in others partially or even wholly destroyed by ulceration. The solitary glands are also often found enlarged, presenting the appearance of a whitish pustule or of a circular excavated ulcer. In some cases all these morbid appearances are observable at the same time in different parts of the same intestine. Such is the fact in regard to a specimen now before me. The mesenteric glands most nearly connected with the ulcerated portions of the intestine are generally enlarged and softened, sometimes being reduced almost to a semi-fluid or creamy consistence. Are these changes in the mucous surfaces and mesenteric glands the result of inflammation or do they arise from that same altered relation between the solids and fluids that gives rise to the enlarged and softened spleen, the engorged lungs &c. &c.? Some of the most eminent investigators of this subject, deny that inflammation has any necessary connection with these changes. Not a few regard the ulcerated patches as resulting from a previous deposit of matter in the glands, its subsequent softening and sloughing away leaving ulcers, much in the same manner that tubercular matter is deposited and subsequently softened in the lungs. Another and perhaps more numerous class of pathologists regard the disease of the aggregated gland of the ilium as specific and peculiar, being strictly analogous to the cutaneous eruptions in that class of diseases styled eruptive fevers. By such the typhoid fever is viewed as a separate and specific disease, to which the disease of Peyer's glands bears a relation very similar to that between the fever and the eruption in Variola. This view is favored by the writings of Louis, Jenner, Bartlett, Gerhard, Flint, &c., and has made a pretty strong impression on the professional mind of this country. Its merits will be more fully considered, however, under the head of Diagnosis and Varieties of Fever. But even the larger portion of this class regard the general softening of the mucous tissue, whether in the stomach or intestines, as not inflammatory, but originating from the same morbid processes which induce the softening in the spleen and other tissues; while the specific affection of the aggregated glands is regarded either as a peculiar inflammation produced by the specific action of the *febrile poison*, or as the result of a primary deposit and its subsequent softening, leaving *atonic* or non-inflammatory ulcers, &c.

Throwing aside for the present, the so-called peculiar affection of Peyer's glands, I think a careful examination of all the facts now accessible to the profession will lead every reflecting reader to the conclusion, that we must give to the word inflammation a very wide and indefinite meaning, or else seek for an explanation of the morbid changes which are apparent, after death from fevers, in the important organs and tissues of the body, in some other process than that of ordinary inflammation. I do not mean to assert that genuine inflammation does not often spring up in the progress of fevers, and become a serious complication, leaving unmistakeable evidence of its existence after death; but simply that the most common post-mortem appearances observed in the viscera of the head, chest, and abdomen, are essentially different from the results of ordinary inflammatory action. And, hence, that the maxim so much insisted on by Dr. Southwood Smith, that "the only morbid condition of fever, of which we have any knowledge, and over which the medical art has any control is that of inflammation," is not true.

Neither will the assertion of Dr. Stokes, that "in the great majority of cases there is local disease of some part or other of the body, and that a vast proportion of fever patients are carried off by LOCAL INFLAMMATION," bear the scrutiny of extended observation. On the contrary, when fevers prevail in their most malignant form, and prove most rapidly fatal, post-mortem examinations reveal the least evidences of local changes of any kind. It is true that when death occurs after a more protracted course of fever, local changes are generally observed in some of the important viscera, but in the large majority of cases these differ essentially, as already detailed, from the well-known results of inflammatory action.

Most of these differences have been stated in connection with the allusions to the changes observed in particular organs, but they may be presented more clearly and forcibly by the following comparison, viz:

| FEVER. | INFLAMMATION. |
| --- | --- |
| 1st.—If my own microscopic observations are correct, or in any degree reliable, both the white corpuscles and fibrin, especially the former, are *diminished* not merely in the general mass of the blood, but equally so in the capillaries of the spleen, lungs, and other tissues presenting evidences of disease after death. | 1st.—One of the most constant and well known effects of inflammation is, the rapid accumulation of *white corpuscles* and *fibrin* both in the general mass of the blood and in the local seat of the disease. |
| 2nd.—The redness so often observed after death from fever | 2nd.—The redness and swelling of an inflamed part are gen- |

| | |
|---|---|
| of membranes of the brain, the mesentery, the mucous surface of the stomach, the spleen, &c., is evidently produced mainly by accumulation of blood in venous capillaries and veins, and is not attended by any increase of white corpuscles in the part. | erally, if not always dependent on accumulation of blood in the arterial capillaries, with effusion or exudation of white corpuscles and liquor sanguinis into the texture of the part affected. |
| 3rd.—All the organs or viscera presenting morbid appearances after death from fever, are *softened* in texture, and very rarely present either plastic exudations constituting false membranes, or purulent matter. | 3rd.—The arterial congestion and infiltration of liquor sanguinis in inflammation tends directly to produce: first, induration or hardness; second, pain; third, suppuration; and fourth, gangrene. |
| 4th—The viscera most commonly affected in fever, such as the spleen, the heart, the mesenteric glands, &c., are generally affected throughout their whole parenchymatous structure alike. Thus, if softening has commenced it generally involves equally the whole thickness of the tissue. This is very unlike ordinary inflammation. | 4th.—When inflammation attacks an organ it seldom if ever affects its whole substance alike, but presents different degrees of progress at different points; thus we find simple congestion, plastic exudation, and suppuration or gangrene, all at different points of the same organ or tissue. |

Such are the more important differences between the changes induced in the solids and fluids by fevers and inflammations; and surely they are sufficient to warrant the inference that the two series of phenomena are not identical either in their origin, progress or final results. What, then, is the true character of the local changes established during the progress of fevers, especially of the continued type? Are they the effects of *congestion*, as stated by Dr. Bartlett, or of Passive Hyperæmia, as it was styled by Dr. Drake? That more or less congestion generally exists in the different organs, chiefly affected, is undoubtedly true. But is this congestion the cause, or merely an accompaniment of the morbid processes set up in the tissues?

That it is the chief cause of the enlargement in those organs that undergo a change of bulk, such as the spleen and glandular structures, there is little room for doubt; but several facts would seem to show, that the important change of texture—the general *softening* of the tissues—which constitutes the characteristic lesion of the solids in almost all fevers, is wholly independent of the congestion. Thus we find portions of the mucous membrane of the stomach and intestines affected with various degrees of softening, from a slight alteration of consistence to complete disorganization without any increased redness or thickness, or other evidence of congestion. The heart, too, is often altered in consistence without any appreciable alteration of size or vascularity. Again, in those organs which present the most marked appearance of congestion and enlargement, like the spleen and mesenteric glands, the alterations of texture are, by no means, in a direct ratio to the degree of apparent congestion, as should be the case if the former resulted from the latter. On the contrary, in many cases where the spleen was most disorganized, it was least changed in size; and M. Louis found the liver softer than natural in one half of the typhoid cases he examined, but in very few was it either enlarged or more vascular than natural. These facts show, not only that neither congestion, passive hyperæmia, nor inflammation, are the *cause* of the alterations of texture commonly found in the solids after death from fever, but they strongly enforce the idea, that if we would comprehend the philosophy of the febrile state, either in its nature, progress, or results, we must proceed a step beyond those morbid conditions called "nervous depression," *congestion* and *inflammation*, which have so long filled the field of vision, and served much the same purpose with pathologists in explaining the nature of disease, that the word *bilious* has with the practitioner in satisfying the too great inquisitiveness of his patients. That nervous depression, congestion, and sometimes inflammation accompany the febrile state, no one doubts.

But that either one, or all of them, combined, constitute essentially the fever, or any particular stage of it, we deny. They are accompaniments and consequences rather than essential ingredients or causes.

Their existence may add to the gravity or fatality of the disease. Nay, their location and extent may render them the chief sources of danger in individual cases; and therefore they are always to be looked for and guarded against by the faithful practitioner.

Whoever has read the preceding pages carefully, (and I presume the number is small who have had the patience to do so) has already perceived two things, viz.:

First, that, in my estimation, fevers, properly so called, are truly idiopathic and general diseases, affecting essentially the solids and fluids of the whole system.

Second, that my views neither coincide with the exclusive *solidist* doctrines of Brown, Darwin, Cullen, Rush, Jackson, and Paine, nor with the modern *humoralist* or chemico-physiological dogmas of Liebig and his followers. That there is, at present, a strong tendency in the profession to a strictly *humoral* pathology; by which I mean a disposition to look to the fluids of the system both for the causes and products of disease, and to strictly chemical changes for an explanation of morbid phenomena. It requires no very profound research to find an adequate reason for this. In the first place, the exclusive solidist doctrines of the last century, were not only found to be erroneous in many respects, but also incapable, by themselves, of explaining many of the physiological and pathological facts constantly being developed. In the second place, the rapid advances made in animal chemistry, and still more, the application of the microscope to physiological researches, leading rapidly to the discovery of many new and important facts, very naturally turned the attention of the investigating portion of the profession strongly in that direction. So much so, indeed, that not a few seem ready to discard all consideration of vital properties in the solids, and seek in the fluids and chemical actions alone the explanation of all the phenomena of health and disease.

This, too, is in strict accordance with the well known tendency of the human mind to vibrate from one extreme to the opposite, whether acting individually or in masses.

The present tendency of medical investigations, however, is not without its advantages. It has already made us acquainted with the composition of the blood and the secretions, as well as with many of the changes they undergo in the varying conditions of health and disease.

They have unfolded to our view, also, the intricate texture and organization of the solids to an extent scarcely thought possible by the generation who preceded the one now on the stage of action. And yet we may say with truth, that the great work of investigation with the test glass and the microscope has only just begun; that there is still a rich and almost boundless field open for investigation, which needs to be cultivated with all the ardor of the most enthusiastic, and yet with the patience and care of the most incredulous. I would by no means discourage investigations, by every means in our power, concerning the composition and qualities of all the fluids of the human body, the changes they undergo in health and disease, and the extent to which they are amenable to ordinary chemical laws. It is only when these investigations are made to occupy the whole mind, to the exclusion of a consideration of all *vital* properties or forces in the solids, that they become objectionable or liable to lead to erroneous conclusions. The great truth, that the solids and fluids of the living animal body, constitute only parts of one whole; that they are so mutually related to each other, that the properties of the one cannot exist without the existence of the other, should be kept constantly in mind. We may push our researches concerning the fluids to the utmost limit of human comprehension, with the application of chemical laws to explain the changes which they undergo, and with that knowledge *alone*, we shall never be able to give a rational explanation of any general disease to which the human system is liable. For, after all our efforts to explain the phenomena of *life* by mere physical laws and forces, we are compelled to come back dissatisfied with our own efforts, and acknowledge that there are in *living* beings *vital properties* or forces, which control, or at least modify the action of all mere physical forces. These *vital* properties seem to be inherent in matter in a state of living *organization*; and hence, belong more especially to the solids, beginning with the most elementary cell that floats in the blood and extending to the most complex organizations. To attempt an explanation of the phenomena of health and disease by a knowledge of the fluids alone and the physical laws and forces which influence them, is like attempting to solve a problem in mathematics without recognizing one of the essential propositions of which it is composed. This is the error of the *humoralist*, whether of ancient or modern times. On the other hand, to attempt a similar task of explanation, by any amount of speculation or reasoning, however logical or acute it may be, concerning the vital properties or forces alone, is like the task imposed on the ancient Israelites by their Egyptian task-masters. It is an attempt to explain phenomena by a knowledge of laws and forces, without an adequate recognition of the materials on which those laws and forces act. This is the error of the exclusive solidist. The fluids of the living animal organization, contain, at once, the materials that supply and the products that proceed from such organization. In a word, they contain the elements that excite, the materials that nourish, and the refuse matter that has served its purpose among the ever-changing atoms of living beings; while the solids contain the vital properties that give direction and character to the whole. In the mutual relations and actions of these lies the true philosophy of life; and in a careful study of these relations, with all the collateral influences that may be made to act on them, are we to seek for the philosophy of disease.

It is objected, perhaps, that this leads us a step beyond the legitimate object of study; that a knowledge of matter as revealed to us in the physical world and the physical laws and forces that govern it, is all that the human mind can comprehend or control; and that *vital* properties and forces are mere imaginary conceptions of which we can know nothing. This objection, however, is based on a false conception of what constitutes our knowledge of inorganic matter and physical forces. It is based on the idea that we know what *matter* and its laws are, on the one hand, and that we propose to know what *vitality* and vital laws are, on the other; neither of which is true. We know nothing of matter in its *essence* any more than we know of the essential nature of vitality. Neither do we propose to investigate either, as they are alike beyond human comprehension.

But we can investigate and know the *properties* of matter and many of the *laws* that govern those properties in their influence on other matter. For instance, we know nothing of the essential nature of electricity; but we do know that there is an agency, which under certain circumstances attracts matter and under others repels it again, and by virtue of this property of attraction and repulsion, the laws governing which become familiar to us, we see that it plays a most important part in the almost infinitely varied phenomena of nature. So, too, we know nothing of the nature of life or vitality; but we do know that all living organized matter possesses a peculiar property which renders it susceptible to the presence of other matter, and gives to that presence actions and changes essentially different from those which take place when the same matter is present with unorganized or dead bodies. To this property we give the name of *vital susceptibility*. To the peculiar arrangement of the particles of matter with which it seems to be inseparably connected, we give the name of *organization*. Of the special agency by which matter is made to assume this organization and manifest this property of susceptibility we know nothing, any more than we know of the essence of that agent that gives rise to the well-known phenomena of electrical attraction and repulsion. But our knowledge of the properties of both is gained in precisely the same manner, namely, by observing their effects and the laws that govern them, and is therefore equally legitimate and equally certain.

The question whether the vital properties depend on an agency, *sui generis*, and independent, or on some modification of the general physical forces, as intimated by Dr. Carpenter, is wholly immaterial, so far as pathological investigations are concerned. For aught we know, light, caloric, electricity, and galvanism may be only different manifestations of one and the same subtle agency; and if this were demonstrated to be the fact it would in no wise alter the relation of these forces to the physical changes which take place in matter. Their several relations and manifestations are so far peculiar that they would require specific names and distinct study as much as in times past. Hence they would still be designated and studied as light, caloric, electricity, &c.

In the same manner, if we should concede, (which I am by no means disposed to do) that vital susceptibility is only a modification or peculiar manifestation of one or more of the ordinary physical forces acting in connection with matter, it would not advance us a single step in either physiological or pathological knowledge. The modification is so great, the manifestation so different from anything exhibited by the physical forces acting in connection with inorganic matter, that a specific and distinctive name would still be required to designate it.

To style that property of living matter which has been called *susceptibility*, caloric, or electricity, would have no other effect than to confuse our ideas and confound our language, without adding an iota to our knowledge.

In studying the great and important class of diseases styled *fevers*, I have endeavored on the one hand to give some clear conception of those properties which were peculiar to and inherent in organized matter, and which I designate as *vital* and elementary, distinguishing them from primary functions as manifested by particular parts; and on the other hand, to note the relations which the fluids of the body and the exterior physical agents bear towards the vital properties of the solids, and the changes which take place in both during the progress of diseases. During this study, I have seen clearly in my own mind, the philosophy of fevers in their development, progress, and results. It has been apparent where they converge to one point, as it were, presenting a certain degree of identity or *oneness*, and yet diverge so far as to present widely different phenomena, and lead to equally different results. Whether I have been fortunate enough to select such language as to convey clearly the ideas I wished to express, remains to be seen. Nevertheless the more obscure topics involved in the general subject will be further illustrated in the next chapter, on the diagnosis and varieties of fever.

